# Comparative genomics of white and opaque cell states supports an epigenetic mechanism of phenotypic switching in *Candida albicans*

**DOI:** 10.1093/g3journal/jkab001

**Published:** 2021-01-22

**Authors:** Chapman N Beekman, Christina A Cuomo, Richard J Bennett, Iuliana V Ene

**Affiliations:** 1 Department of Molecular Microbiology and Immunology, Brown University, Providence, RI 02912, USA; 2 Infectious Disease and Microbiome Program, Broad Institute, Cambridge, MA 02142, USA

**Keywords:** *Candida albicans*, epigenetic switch, comparative genomics

## Abstract

Several *Candida* species can undergo a heritable and reversible transition from a ‘white’ state to a mating proficient ‘opaque’ state. This ability relies on highly interconnected transcriptional networks that control cell-type-specific gene expression programs over multiple generations. *Candida albicans*, the most prominent pathogenic *Candida* species, provides a well-studied paradigm for the white-opaque transition. In this species, a network of at least eight transcriptional regulators controls the balance between white and opaque states that have distinct morphologies, transcriptional profiles, and physiological properties. Given the reversible nature and the high frequency of white-opaque transitions, it is widely assumed that this switch is governed by epigenetic mechanisms that occur independently of any changes in DNA sequence. However, a direct genomic comparison between white and opaque cells has yet to be performed. Here, we present a whole-genome comparative analysis of *C. albicans* white and opaque cells. This analysis revealed rare genetic changes between cell states, none of which are linked to white-opaque switching. This result is consistent with epigenetic mechanisms controlling cell state differentiation in *C. albicans* and provides direct evidence against a role for genetic variation in mediating the switch.

## Introduction

The ability to generate and maintain diverse cell types is a central theme in biology. Eukaryotic cell fate is often regulated by epigenetic mechanisms that do not involve a change in the primary DNA sequence. Instead, heritable changes are defined by gene expression programs driven by transcription factor networks that act in combination with alterations in histone and DNA modifications (Christophersen and Helin 2010; Rhee and Ho 2017). Autoregulation of the transcription factors within these networks is a key feature of cell fate determination that enables stable propagation of cell states (Crews and Pearson 2009).

Although epigenetic mechanisms have been extensively studied in multicellular eukaryotes, single-celled eukaryotes can also undergo epigenetic transitions between heritable cell states. This is thought to occur in several *Candida* species that have the ability to reversibly transition between alternative ‘white’ and ‘opaque’ states (Slutsky *et al.* 1987; Pujol *et al.* 2004; Porman *et al.* 2011; Xie *et al.* 2012). The white-opaque switch has been most extensively studied in *Candida albicans*, a commensal fungus of the human gastrointestinal tract (GI) but also a prevalent opportunistic pathogen *(**Pappas et al.* 2018). Here, formation of the opaque state is governed by a complex network of at least eight transcriptional regulators (Srikantha *et al.* 2000; Zordan *et al.* 2006, 2007; Wang *et al.* 2011; Hernday et al. 2013; Lohse *et al.* 2013; Hernday *et al.* 2016; Lohse *et al.* 2016). Switching between white and opaque states occurs stochastically and is also driven by multiple environmental cues including temperature, CO_2_ and N-acetyl glucosamine (Rikkerink *et al.* 1988; Ramírez-Zavala *et al.* 2008; Huang *et al.* 2009; Alby and Bennett 2009a, 2009b; Huang *et al.* 2010).

*Candida albicans* white and opaque states display distinct cellular morphologies (opaque cells are elongated relative to the more spherical white cells), and are named for their colony appearance as opaque colonies are darker and flatter than brighter, dome-shaped white colonies (Slutsky *et al.* 1987). In addition to morphological differences, white and opaque cells show stark differences in their mating ability, with opaque cells being able to undergo mating a million times more efficiently than white cells (Miller and Johnson 2002; Scaduto *et al.* 2017). White and opaque cells also differ in their metabolic capacity (Lan *et al.* 2002; Ene *et al.* 2016), drug susceptibility (Ene *et al.* 2016), interaction with immune cells (Sasse *et al.* 2013; Mallick *et al.* 2016), and adherence to host tissue (Kennedy *et al.* 1988). Accordingly, these cell types display differing host niche preferences, with white cells displaying increased relative fitness across multiple niches, whereas opaque cells appear better adapted for colonization of the skin (Kvaal *et al.* 1999; Pande *et al.* 2013; Noble *et al.* 2017; Takagi *et al.* 2019).

Given the importance of the white-opaque switch to *C. albicans* biology, its regulation has been a major research focus. *Candida albicans* is typically a heterozygous diploid species and initial studies indicated that a homozygous mating locus (*MTL***a**/**a** or alpha/alpha) is a prerequisite for white-to-opaque switching (Miller and Johnson 2002). In *MTL* homozygous strains, white-to-opaque switching occurs every 10^4–^10^5^ generations under standard laboratory conditions (Rikkerink *et al.* 1988). More recently it was shown that *MTL* heterozygous (*MTL***a**/alpha) strains can also undergo the white-opaque switch under select conditions (Xie *et al.* 2013; Sun *et al.* 2016). Switching is highly regulated at the transcriptional level via a network of interconnected transcriptional regulators including Wor1, Wor2, Wor3, Wor4, Ahr1, Ssn6, Efg1, Czf1 (Srikantha *et al.* 2000; Huang *et al.* 2006; Srikantha *et al.* 2006; Zordan *et al.* 2006; Vinces and Kumamoto 2007; Zordan *et al.* 2007; Lohse *et al.* 2013; Hernday *et al.* 2016; Lohse *et al.* 2016). Multiple positive feedback loops between these transcription factors contribute to the stable inheritance of cell states (Zordan *et al.* 2007; Hernday *et al.* 2013).

Currently, the white-opaque switch is presumed to be epigenetic, yet a genomic comparison of white and opaque cells has yet to be conducted, despite examples of genetically regulated cell state transitions in other microbial species (van der Woude 2011; Darmon and Leach 2014; Norman *et al.* 2015). This question is also relevant given the high levels of genomic plasticity observed in *C. albicans* strains (Forche *et al.* 2009, 2011; Bennett *et al.* 2014; Hirakawa *et al.* 2015; Todd *et al.* 2019). The recent discovery of a mutational transition in *C. albicans* driven by loss-of-function mutations in a known white-opaque regulator (Liang *et al.* 2019) also illustrates how genetic changes can generate distinct phenotypic states that could be mistaken for epigenetic switching.

Here we present the first comparative genomic analysis of white and opaque *C. albicans* isolates using deep Illumina whole-genome sequencing. We conducted a comprehensive analysis of copy number variants, structural variants (SVs), loss of heterozygosity events and point mutations. Limited genetic differences were identified between cell states from matched white/opaque cells, and these differences were not shared across lineages nor were they located in genes with known roles in white-opaque switching. These findings are consistent with the current paradigm that white-opaque switching is epigenetic and occurs without genomic alterations.

## Materials and methods

### Strain construction and selection of white and opaque colonies

*Candida albicans* strains were derived from isolate SC5314 either by targeted deletion of the *MTL*alpha locus generating strain A (*MTL***a**/-), or via sorbose-mediated selection to generate strain B [RBY1118 (**a**/**a**) (Schaefer *et al.* 2007)]. SC5314 was transformed with plasmid pRB102 (Sherwood and Bennett 2008) to delete *MTL*alpha and resulting in integration of the *SAT1* gene (*SAT1*^R^). Correct integration was checked by PCR using oligos 51–54 as previously described (Alby and Bennett 2009a). Colonies were grown in YPD (2% peptone, 1% yeast extract, 2% glucose, 25 μg/ml uridine) to allow excision of the *SAT1* cassette and plated on YPD plates supplemented with nourseothricin (25 μg/ml) to identify *SAT1*^S^ colonies. Excision of the *SAT1* cassette was verified by PCR using oligos 51–54 (Alby and Bennett 2009a), giving rise to strain A.

Opaque cells of A and B isolates were obtained from glycerol stocks of pure opaque populations by streaking out for single colonies on SCD solid medium [0.7% yeast nitrogen base without amino acids, 2% glucose, amino acid mix (uracil, uridine, histidine, leucine, arginine)] and growing for six days at room temperature. For each lineage, a full opaque colony was re-streaked onto a fresh SCD plate and allowed to grow for six days, at which point colonies were inspected for switching to the white state. A full opaque and a full white colony (indicating that cells formed from a recent opaque-to-white switch) were harvested from this plate and grown overnight (∼18 h) in liquid SCD for genomic DNA extraction and microscopy. Only cultures displaying a minimum of >95% purity in cell identity were used for subsequent analysis.

### Microscopy

Selected white and opaque colonies were removed with a toothpick from solid SCD media, mixed with sterile H_2_O and wet-mounted on glass slides. Cells were imaged using differential interference contrast (DIC) on a Zeiss Axio Observer Z1 inverted microscope.

### Whole-genome sequencing

To extract genomic DNA, white and opaque cells were grown overnight in SCD liquid media at 25° and DNA was isolated from ∼10^9^ cells using a Qiagen Genomic Buffer Set and a Qiagen Genomic-tip 100/G kit according to manufacturer’s instructions. Libraries were made using the Nextera XT DNA Library preparation kit protocol (Illumina) with an input of 2 ng/μL in 10 μL. Each isolate was sequenced on an Illumina HiSeq X generating 101 bp paired reads. The nuclear genome sequences and General Feature Files (GFF) for *C. albicans* SC5314 reference genome (version A22-s07-m01-r69) were obtained from the *Candida* Genome Database (www.candidagenome.org). Reads were aligned to the SC5314 reference genome (haplotype A chromosomes) using Burrows-Wheeler Aligner (BWA) v0.7.4-r385 mem (Li and Durbin 2009), and converted to sorted BAM format using Samtools v0.1.9 (r783) (Li *et al.* 2009). Briefly, reads were trimmed using trimmomatic 0.36 (with default parameters except for slidingwindow: 10:25 and minlen: 75) (Bolger *et al.* 2014) and Picard Tools (http://broadinstitute.github.io/picard/). AddOrReplaceReadGroups, MarkDuplicates, CreateSequenceDictionary and ReorderSam were used to preprocess the alignments. We used GATK4 (Brouard *et al.* 2019) RealignerTargetCreator and IndelRealigner for resolving misaligned reads close to indels on parental-progeny pairs of isolates to avoid discrepancies between isolates. This resulted in an average of 16.76 (SD ± 5.79) million paired reads and 166.7X (SD ± 57.5X) coverage per sample.

### Variant calling and validation

The Genome Analysis Toolkit (GATK4) (McKenna *et al.* 2010; Brouard *et al.* 2019) was used to call both variant and reference bases from the alignments. GATK Haplotype Caller and Pilon (Walker *et al.* 2014) (with diploid genotyper ploidy setting) were run with both SNP and INDEL genotype likelihood models (GLM). We then merged and sorted all the calls from Haplotype Caller and ran VariantFiltration with the following filters QD < 2.0, FS > 60.0, MQ < 40.0, MQRankSum < –12.5, ReadPosRankSum < −8. Next, we removed any base that had less than a minimum genotype quality (QUAL) of 50, or a minimum read depth (DP) of 20. Finally, we removed any positions that were called by both GLMs (*i.e.*, incompatible indels and SNPs), any marked as “LowQual” by GATK, nested indels, or sites that did not include a PASS flag. Similar filtering was performed for Pilon calls, removing low-quality sites and setting a minimum read depth of 20. A minimum allelic frequency difference of 25% between compared genome pairs was used to establish mutations. All mutations identified between white and opaque strains were visually inspected using IGV (Robinson *et al.* 2011).

GATK4 outputs were used to calculate allele frequencies for each heterozygous position across all eight *C. albicans* chromosomes. Thus, all heterozygous positions identified in the reference strain (SC5314 A22) were examined across isolates and the percent of reads mapping to each chromosome homolog was determined for each heterozygous position. Allele frequencies for each chromosome homolog were plotted using GraphPad Prism 8. To identify large LOH events, we calculated the number of heterozygous positions per 1 kbp region and averaged across 10 kbp windows (Figure 2A, red lines). Average heterozygosity levels for each isolate were plotted using GraphPad Prism 8.

### Ploidy and copy number variation analysis

To examine copy number variation across the genome, the Illumina read alignment depth was calculated for 1 kbp windows across the genome using BEDTools 2.28 (Quinlan and Hall 2010) and SAMtools 1.3.1 (Li *et al.* 2009). Read depths were calculated as the number of bases aligned per window divided by the length of the window (1000 bp or size of coding regions) and normalized to the average read depth for each genome and by GC content using pybedtools 0.8.1 (Quinlan and Hall 2010; Dale *et al.* 2011). The normalized alignment depth for each 1 kbp was averaged across 10 kbp for plotting (GraphPad Prism 8) and analysis of large-scale CNV. Both 1 kbp windows and chromosomal elements were examined for small CNV. Chromosomal elements examined included 6213 coding regions (ORFs), 156 tRNAs, 75 snoRNAs, six rRNAs, five ncRNAs and five snRNAs, as defined by the *Candida* Genome Database for SC5314 A22. Regions were considered to be a site of CNV if coverage for a region on the opaque state genome was two standard deviations above or below the coverage determined for the corresponding region of the white state genome.

### SV calling

SVs were called using Pindel 0.2.0 (Ye *et al.* 2009) with potential variants including inversions, deletions, insertions, duplications and replacements. Pindel was ran with default options setting and -M 10 as previously described (Ghoneim *et al.* 2014). All variants called were filtered based on allele frequency differences between the two cell types and those showing differences higher than 25% were visually inspected using IGV.

### Data availability

Strains are available upon request. Data generated under this study can be found under NCBI BioProject PRJNA686122, including whole-genome assemblies and respective annotations for white and opaque isolates. Raw reads for the parental isolate SC5314 have been previously published under NCBI BioProject PRJNA193498 (Hirakawa *et al.* 2015). Supplementary material includes Supplementary Table S1, which contains additional details on CNV analysis (uploaded to figshare: https://doi.org/10.25387/g3.13501908).

## Results and discussion

### Sequencing of *Candida albicans* white and opaque isolates

**Figure 1:**
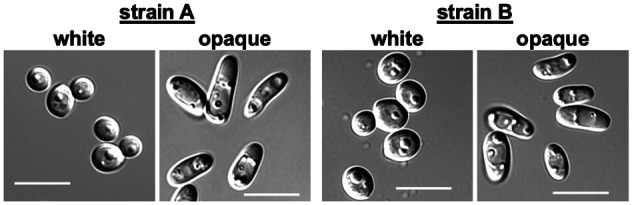
Cell morphologies of *Candida albicans* white and opaque cells. Cells were obtained from SC5314 derivatives that are *MTL***a**/- (lineage A1) or *MTL***a**/**a** (lineage B1). Scale bars, 10 μm.

We generated *MTL* hemizygous or *MTL* homozygous derivatives of *C. albicans* strain SC5314 (*MTL***a**/alpha) by deletion of the *MTL*alpha locus (isolate A; *MTL***a**/-) or via sorbose selection and outgrowth on YPD medium [isolate B; *MTL***a**/**a** (Schaefer *et al.* 2007)]. The derived strains undergo stochastic switching between the white and opaque cell states. Opaque cells of each strain were inoculated on SCD medium and grown for six days at 22°. From this plate, a single opaque colony was restruck on SCD and again grown for six days at 22°. A subset of colonies presented in the white state (*i.e.*, had switched to the white state from the opaque state) based on colony appearance and cellular morphology. Cells from opaque colonies were larger and more elongated whereas cells from white colonies were smaller and more spherical, as expected (Figure 1). Verified white and opaque colonies from each strain were cultured overnight (∼18 h) and genomic DNA isolated. Using this strategy, any mutations identified between white and opaque cells would have occurred either during the 12 days of growth on SCD or during liquid culturing prior to DNA isolation. In addition, we performed these experiments in duplicate, generating independent, paired white/opaque versions for isolate A (lineages A-1 and A-2) and isolate B (lineages B-1, B-2).

DNA from white and opaque cells was sequenced on the Illumina HiSeq X platform using 101 bp paired end reads. Reads from each genome (white and opaque forms for A-1, A-2, B-1, and B-2) were aligned to the SC5314 reference genome (assembly 22) with an average of 98.29% (SD ± 0.52%) of paired reads successfully mapped across the genome reference, and an average of 99.87% (SD ± 0.017%) of the reference genome covered by reads. This resulted in average coverage levels of 166.7X (SD ± 57.5X) across the sequenced white and opaque cell genomes.

### Karyotype and heterozygosity levels are similar between white and opaque cell genomes

**Figure 2:**
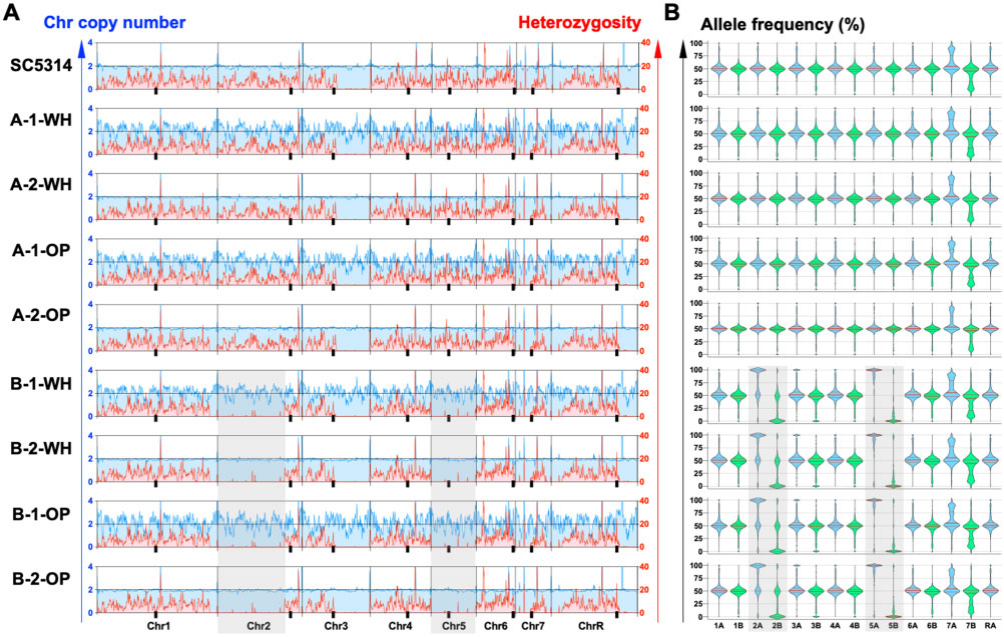
Chromosome copy numbers and heterozygosity levels for white and opaque isolates. (A) Coverage was normalized to disomic levels and relative read depth was plotted across all eight *Candida albicans* chromosomes to determine their relative copy numbers, plotted in blue. The number of heterozygous positions was examined for each chromosome and average heterozygosity per 10 kbp window is plotted in red. Lines and tick marks denote chromosomes and centromere positions, respectively, on each chromosome. Shaded boxes indicate LOH regions unique to the B-1 and B-2 lineages. The parental strain SC5314 is included for reference. WH, white state; OP, opaque state. (B) Allele frequencies were determined for white/opaque cells and parental strain SC5314. Frequencies were compared across chromosomes as indicated for homolog A (blue) and homolog B (green) for each isolate. Note that allele frequencies have a median value of ∼50% for heterozygous disomic chromosomes, whereas median values are skewed toward 0% or 100% for chromosomes that are homozygous. Center lines represent median frequencies, upper and lower lines indicate 25th and 75th percentiles, shaded boxes indicate LOH regions unique to the B-1 and B-2 lineages.

As aneuploidy and large losses of heterozygosity (LOH) events can frequently arise in diploid *C. albicans* isolates following transformation (Bouchonville *et al.* 2009) or sorbose selection (Hickman *et al.* 2015), we first examined the sequenced genomes for evidence of such large-scale events. Mean coverage levels were calculated per 10 kbp regions across the eight chromosomes and normalized by the mean read depth for each genome (Figure 2a, blue lines). This analysis indicated that the evaluated genomes showed disomic levels across all eight chromosomes in each case.

LOH events involve the loss of genetic information from one homolog across a segmental region or even a whole chromosome. This can occur either by deletion/loss of a chromosomal region or via recombination between homologs (*e.g.*, gene conversion or break-induced replication) and is detected by the loss of heterozygous positions across the impacted region. To identify LOH events, heterozygous positions were mapped using GATK and the number of heterozygous positions calculated for each genome using 1 kbp regions and averaged across 10 kbp windows (Figure 2A). This revealed homozygous regions across the right half of chromosomes (Chr) 3 and R in all eight analyzed genomes, regions that are also homozygous in the parental SC5314 lineage (Figure 2A) (Hirakawa *et al.* 2015). Additional LOH regions in the B1 and B2 lineages included the whole of Chr 5 as well as the left arm of Chr 2 (Figure 2A). LOH of Chr 5 is likely a result of using sorbose selection to generate the *MTL* homozygous isolate B, as growth on this medium frequently results in the loss of one homolog of Chr 5 (Kabir *et al.* 2005; Andaluz *et al.* 2007; Hickman *et al.* 2015). Importantly, however, genomes from white/opaque pairs displayed matching patterns of heterozygosity indicating that large LOH events did not accompany the white-opaque switch.

Mean allele frequencies for each chromosome were compared to those in the parental SC5314 genome, as these can reveal both LOH and changes in chromosome copy number. The mean frequencies were close to 50% for most chromosomes, consistent with these chromosomes being disomic and heterozygous in the evaluated genomes (Figure 2B). B-1 and B-2 lineages (both white and opaque cells) displayed mean allele frequencies close to 0% and 100% for Chr 2 and 5, respectively, consistent with large LOH events having occurred in the sorbose-selected parent of these lineages. Overall, however, no notable differences in allele frequencies were observed between genomes from matched white/opaque pairs, establishing similar heterozygosity levels between the two cell states.

### Rare single nucleotide variants exist between matched white and opaque cells

**Figure 3:**
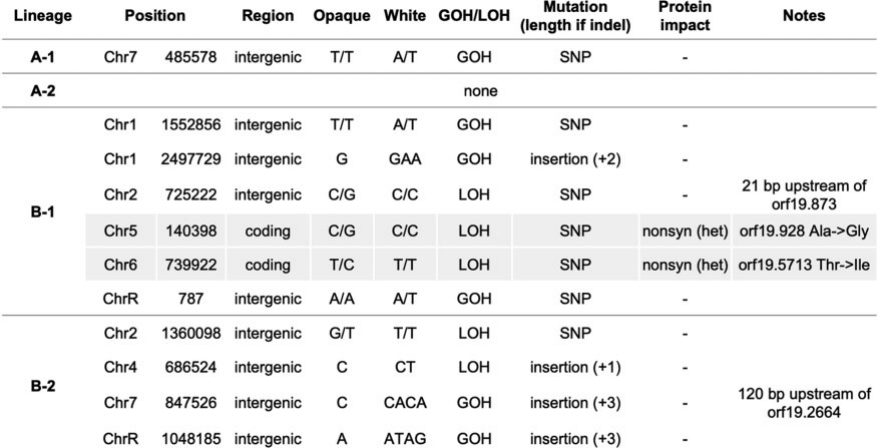
Differences in DNA sequence identified between paired white and opaque cells of each lineage. Mutations were identified with either GATK or Pilon and were inspected in IGV. For mutations identified in close proximity to ORFs, the distance relative to the respective gene is specified. LOH, loss of heterozygosity; GOH, gain of heterozygosity; SNP, single nucleotide polymorphism; nonsyn, nonsynonymous mutation. Shaded rows indicate nonsynonymous mutations.

To assess small-scale variants such as single nucleotide polymorphisms (SNPs) and indels (insertions/deletions) that may have occurred during the opaque-to-white transition, variants distinguishing white/opaque states were identified using GATK (McKenna *et al.* 2010) and Pilon (Walker *et al.* 2014), and visually confirmed using IGV. This revealed one mutation (1 SNP) between A-1 white/opaque cells and no mutations between A-2 white/opaque cells. For the B lineages, six mutations (one insertion and five SNPs) were identified between B-1 white/opaque cells and four mutations (one SNP and three insertions) were identified between B-2 white/opaque cells (Figure 3). None of these mutations were shared across multiple lineages. For B-1 and B-2 lineages, these mutations involved an equal number of LOH events and *de novo* base substitutions, the latter resulting in a gain of heterozygosity, or GOH, event. This is consistent with previous reports that LOH and GOH events often occur at similar frequencies during *C. albicans* microevolution (Ene *et al.* 2018). All mutations with the exception of two SNPs in the B-1 white/opaque comparison were located in intergenic regions. None of the identified indels therefore resulted in frameshifts. The two B-1 mutations located in coding regions were the result of short-tract LOH events that produced nonsynonymous mutations, thereby leading to changes in the encoded amino acid in only one chromosome homolog. One of the genes affected is an uncharacterized open reading frame (ORF, orf19.928), whereas the other is a putative NADH dehydrogenase based on sequence homology (orf19.5713, *YMX6*). None of the genes impacted by nonsynonymous mutations or located proximal to intergenic mutations (orf19.873 and orf19.2664, Figure 3) have any known connections to white-opaque switching and are not differentially expressed between the two cell states (Tuch *et al.* 2010), yet further investigation may be warranted to fully rule out a role for these genetic changes in switching. Similarly, a search for structural variants including indels, inversions, duplications and replacements using Pindel (Ye *et al.* 2009) did not identify any variants between matched white/opaque pairs, indicating that this type of structural events are not associated with white-opaque switching.

### Copy number variation between white/opaque states

**Figure 4:**
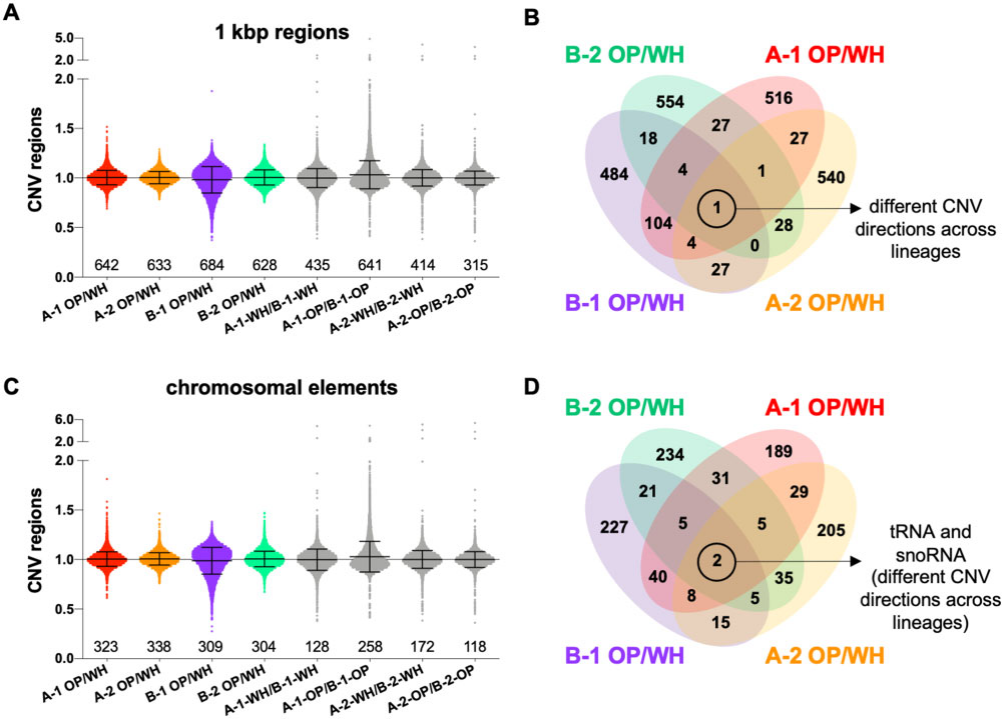
Copy number variation (CNV) between white and opaque cells. CNVs were identified using read coverage depth across 1 kbp windows (A) and different chromosomal elements (C) for the four A and B lineages (using two standard deviations above or below coverage levels as cutoffs for CNV). Comparisons were made between white/opaque states within a lineage and between A and B lineages for cells of the same type within the same sequencing run. Numbers indicate the total number of CNV regions identified in each comparison (above or below two standard deviations), lines indicate means and standard deviations. OP, opaque; WH, white. Venn diagrams indicate the number of shared CNVs identified between cell states for 1 kbp windows (B) and for chromosomal elements (D). Control comparisons between lineages as well as a full list of the common regions along with corresponding *Candida albicans* genome annotations are included in Supplementary Table S1.

Copy number variation (CNV) has emerged as a powerful strategy for genetic variation and recent yeast studies have shown that amplification of particular gene regions can drive rapid adaptation to stress (Todd and Selmecki 2020) or nutrient limitation (Lauer *et al.* 2018). These CNVs are transient so that cells can revert after removal of the selective pressure (Todd and Selmecki 2020). This raises the possibility that a similar mechanism could enable the white-opaque switch. To look for CNV between genomes from white/opaque cells, we examined differences in coverage across both 1 kbp windows and chromosomal elements including gene coding regions and small RNAs (tRNAs, rRNAs, ncRNAs, snoRNA and snRNAs). We identified a small number of regions showing CNV between white and opaque cells from the same lineage, as well as CNV regions when comparing cells of each type from different lineages (Figure 4, A and C). Regions displaying elevated or reduced copy number were compared between the four lineages using two standard deviations as a cutoff. This comparison identified one 1 kbp region and two small RNAs (one tRNA and one snoRNA) as having differential coverage in white/opaque pairs in all four lineages (Figure 4, B and D). However, none of these CNV regions showed consistent directionality (elevated or decreased) with respect to coverage levels in white v. opaque cells (Supplementary Table S1). Additional CNV comparisons were carried out between genomes of the same cell type but different lineages from the same sequencing run (*e.g.*, A-1 white *vs* B-1 white). This analysis identified 12 1 kbp regions and 46 chromosomal elements that were common across the four comparisons (Supplementary Table S1). In contrast to comparisons between cell states, this analysis revealed consistent CNV directionality and multiple lineage-specific differences, thus validating these CNV regions. Lineage-specific CNVs included those associated with sorbose selection, differences in auxotrophic makers (*HIS1*, *URA3*), differences in *MTL*a copy number (reflecting *MTL*a/- in the A-1 and A-2 lineages versus *MTL*a/a in the B-1 and B-2 lineages), as well as CNVs associated with repeat regions (Supplementary Table S1). Although further investigation of these CNV regions may be warranted, the limited number of regions identified as well as the lack of consistent directionality in coverage levels between white and opaque cell genomes do not point to a strong link between CNV and white-opaque switching.

Here, we provide a comparative genomic analysis of *C. albicans* white and opaque cells. The lack of consistent genetic changes between the two cell states (including changes in karyotype, copy number, structural variants or point mutations) is in line with the current epigenetic model for regulation of white-opaque switching. Although we cannot rule out a role for the genetic variants identified here in contributing to switching or to phenotypic differences between states, we consider this unlikely given that genetic changes were not shared by the independent lineages examined here, as well as the lack of a clear pattern for CNVs that present in all lineages.

## Supplementary Material

jkab001_Supplementary_Data
